# Associations of socioeconomic and other environmental factors with early brain development in Bangladeshi infants and children

**DOI:** 10.1016/j.dcn.2021.100981

**Published:** 2021-06-18

**Authors:** Sarah K.G. Jensen, Wanze Xie, Swapna Kumar, Rashidul Haque, William A. Petri, Charles A. Nelson

**Affiliations:** a Department of Pediatrics, Boston Children’s Hospital, Boston, MA, USA; b Department of Pediatrics, Harvard Medical School, Boston, MA, USA; c Boston College School of Social Work, Boston College, MA, USA; d Icddr,b, Dhaka, Bangladesh; e University of Virginia, Infectious Diseases & International Health, Charlottesville, VA, USA; f Harvard Graduate School of Education, Cambridge, MA, USA

**Keywords:** EEG, electroencephalography, Poverty, EEG, Development, Stress, Caregiving, Bangladesh, Low and middle income country

## Abstract

Studies of infants growing up in high-income countries reveal developmental changes in electroencephalography (EEG) power whereby socioeconomic factors - specifically, low SES and low income - are associated with lower EEG power in infants aged newborn to nine months. In the current paper we explore relationships of spectral EEG power across three regions (frontal, central, and parietal) and four frequency bands (theta, alpha, beta, and gamma) with socioeconomic and psychosocial factors in a cohort of n = 160 6-month-old infants and n = 187 36-month-old children living in Dhaka, Bangladesh. Household wealth is assessed as a multi-dimensional composite score encompassing income, assets, and housing materials. Psychosocial factors include maternal perceived stress and family caregiving activities. Among the 6-month-old infants we do not observe any association of household wealth or psychosocial factors with EEG power. Among the 36-month-old children, we find that household wealth is negatively associated absolute power in the beta and gamma bands across frontal, central, and parietal electrodes. We also find that higher reports of maternal perceived stress are associated with more absolute theta power in frontal and central regions in the 36-month-old children. The finding of a negative relationship of household wealth with beta and gamma power in 36-month-old children differs from findings previously observed in infants in high-income countries. Overall, findings suggest that children’s environment continues to influence the development of EEG oscillations and provide support for the utility of EEG to quantify developmental effects of early life experiences on neural functional outcomes in low income countries.

## Highlights

• Research from high-income countries shows that experiences impact neural development.
• We examine EEG in relation to wealth and psychosocial factors in 6 and 36-month-olds from Bangladesh.
• Wealth and maternal stress is associated with EEG oscillations in 36-month-olds.
• Neither wealth nor maternal stress is associated with EEG oscillations in 6-month-olds.
• Environmental influences on EEG may emerge between 6 and 36 months of age.

## 1 Introduction

Environmental influences on child development are well-known and behavioral studies from throughout the world show that children living in impoverished circumstances tend to reach developmental milestones later than children living in wealthier families. Indeed, a large body of literature from both high and low-income countries has shown that children in poor households perform less well on measures of cognitive functions and language compared with wealthier peers (Jensen et al., 2019b, c; Johnson et al., 2016; Rubio-Codina and Grantham-McGregor, 2019). Emerging evidence from longitudinal research suggests that socioeconomic disparities in child development emerge sometime during the first two years of children’s life. For example, using data from Bangladesh we have previously shown a pattern whereby children perform as expected with regards to developmental milestones at 6 months, but have dropped significantly at 27 months (Jensen et al., 2019b). We have also shown that poverty predicts child outcomes via exposure to risks such as poor growth, inflammation, and playful parenting from 27 months onwards, while only playful parenting showed an association with milestone related to language at 6 months (Jensen et al., 2019c). We previously proposed that these findings likely reflect one of three possibilities: 1) a developmental effect whereby children gradually fall behind on developmental milestones over time due to an acceleration of developmental milestones achieved with age, 2) a cumulative effect whereby exposure to poverty-related risks accumulate over time such that effects only emerge after a certain amount of “environmental exposure” to poverty, or 3) a methodological confound in behavioral assessments whereby the ability of developmental assessments to differentiate between children’s developmental status may improve as children age given the broader range of tasks children can perform as they age. In this study we use electroencephalography (EEG) to examine associations of socioeconomic and psychosocial factors with an objective measure of neurofunctional outcomes in 6-month-old infants and 36-month-old children. The aim of this paper is to shed further light at the developmental timing whereby neurological correlates of adverse experiences begin to emerge.

Neuroimaging provides an important lens with which to study possible neurodevelopmental mechanisms underlying environmental influences on child development because the neurological correlates of poverty, which are broadly expected to underlie well-characterized effects of poverty on children's behavioral outcomes, would be expected to emerge earlier than behavioral effects of poverty can be detected. A handful of studies have examined EEG spectral power over the scalp as a potential biomarker of socioeconomic influences on brain development (Brito et al., 2016; Pierce et al., 2019; Tomalski et al., 2013). Electroencephalography is a non-invasive, relatively inexpensive and easy-to-use tool compared with other neuroimaging methodologies, and these advantages make it a well-suited measure for neurodevelopmental studies among vulnerable populations. Electroencephalography can be implemented in low resource environments as evidenced by previous work in Bangladesh (Jensen et al., 2019a; Xie et al., 2019a, b) and other low-income countries such as Pakistan (Tarullo et al., 2017) and The Gambia (Katus et al., 2020). Electroencephalography records electrical signals distributed across the scalp surface. These signals reflect the synchronous activity of cortical (pyramidal) neurons which, in turn, can be decomposed into neural oscillations in different frequency bands that are believed to make distinct and important contributions to cognitive processes (Bell and Cuevas, 2012). For example, the magnitude of spectral power in the theta, alpha, and beta bands in children’s EEG has been associated with sustained attention and error monitoring in children (Xie et al., 2018; Buzzell et al., 2020), while the magnitude of gamma power has been found to be consistently associated with language abilities (Brito et al., 2016; Gou, Choudhury, et al., 2011; Cantiani et al., 2019).

Previous studies have found that patterns of spectral EEG power across different frequency bands may serve as an index of brain maturation. For example, developmental studies using mostly cross-sectional data from high-income countries have described a developmental pattern of resting-state EEG power whereby typically developing children display a concurrent developmental decrease in low-frequency rhythms (i.e., delta and theta) and increase in high-frequency rhythms (i.e., alpha, beta, and gamma) throughout childhood (Saby and Marshall, 2012). The sensitivity of EEG to detect fine-grained developmental change positions EEG as an ideal measure for detecting whether neural maturation shows early signs of derailment from a healthy developmental trajectory. Indeed, studies exploring possible neurodevelopmental markers of socioeconomic and other environmental factors suggest that the above mentioned normative developmental shift from an EEG profile dominated by a higher magnitude of low frequency power towards a more “mature” profile characterized by a higher magnitude of high frequency power may be influenced by environmental factors. Environmental factor that have been found to explain variation in children’s EEG power profile include household income (Tomalski et al., 2013), socioeconomic status (Cantiani et al., 2019), maternal stress (Pierce et al., 2019), and early psychosocial deprivation (Marshall et al., 2008). Overall, previous findings suggest that infants and children who have been exposed to socioeconomic and/or psychosocial risks exhibit a lower magnitude of absolute and/or relative high frequency power, compared with children who have had lower risk exposure. A study from the United States, for instance, found that environmental influences on EEG emerged within the first 2 months of life in that absolute beta and gamma power was positively associated with maternal education, and negatively associated with maternal perceived stress in 2-month-old infants (Pierce et al., 2019). These findings are largely in line with findings from the United Kingdom where infants from low-income households were found to exhibited lower absolute gamma power compared with infants from wealthier households at 6–9 months of age (Tomalski et al., 2013), and Italy where higher socioeconomic status was found to be associated with gamma power in 6-month-old infants (Cantiani et al., 2019). Associations of biological measures of maternal stress (hair cortisol) with children’s EEG power have also been observed in a study from the United States, which found that higher maternal cortisol concentrations in hair (reflecting higher levels of physiological stress within the past 3 months) were associated with higher levels of relative theta and lower levels of relative beta power in 6 to 12-month-old infants (Troller-Renfree et al., 2020). A study of neonates from the United States, however, did not show association of household or neighborhood factors with infant EEG power (Brito et al., 2016), suggesting that such effects emerge during development.

Few studies have examined EEG outcomes in relation to exposures to socioeconomic and psychosocial risks among infants and children growing up in low- and middle-income countries. Studies of 18 to 30-month-old (Otero, 1994) and 48-month-old (Otero, 1997) children from Mexico have described a pattern whereby children from low-income homes showed an EEG power profile of more low frequency power compared with high frequency power, largely similar to what has been observed in high income countries.

Wealth, generally considered a multidimensional construct, likely affects neural development through multiple factors including nutrition and health as well as psychosocial factors such as caregiver well-being and engagement in responsive caregiving activities. Indeed, we know that in low-income countries, like Bangladesh. factors that commonly correlate with wealth include access to nutrition, material resources (including toys and books), sanitation and access clean water. Moreover, it is broadly recognized that poverty is a key source of stress in both parents (Ursache et al., 2017) and children (Lupien et al., 2001) and that poverty-related stress may have profound influence on the psychosocial caregiving environment, thereby exerting long-term effects on children’s opportunities to thrive. Parental stress likely mediate effects of poverty on children’s cognitive development via the impact of stress on caregivers’ ability to engage in responsive care - a known mediator of poverty-related effects on child development as shown in studies from diverse geographical settings including the United States (Hackman et al., 2015), Colombia (Rubio-Codina and Grantham-McGregor, 2019), and Bangladesh (Jensen et al., 2019c). Neuroimaging studies using magnetic resonance imaging (MRI) have also shown that the quality of the caregiving environment mediates the adverse effects of poverty on children’s neurological outcomes (Luby et al., 2013). While both parental wealth and education may influence children’s neurodevelopmental outcomes, we hypothesize that they operate along somewhat distinct pathways. The effect of low wealth (poverty) are likely to be mediated by a wide array of environmental factors such as food insecurity, resources deprivation, and poor hygiene, as well as social factors related to parental stress and caregiving activities. Poor maternal education, while often correlated with poverty, may in itself mostly affect children development through reduced access to information about child care and stimulation.

The purpose of this study was to examine associations of household wealth with EEG power in 6- and 36-month-old infants living in the Mirpur district of Dhaka, an area that encompasses both very low-income and middle-class neighborhoods. We explore two hypotheses, namely that household wealth will be positively associated with absolute high-frequency EEG power (beta and gamma). We further hypothesize that we will see a temporal lag whereby effects may not be observed in 6-month-old infants, but in 36-month-old children (Jensen et al., 2019a). We also hypothesize that the associations of household wealth with EEG power will be partially accounted for by maternal stress and family caregiving activities. We add to the current literature by having recruited a unique sample of infants and children from an urban slum area in Bangladesh - a population that continues to be significantly underrepresented in neurodevelopmental research (Jensen et al., 2019b). We use EEG to assess possible objective neurological correlates of early life experiences in a low-income country where poverty and related adverse exposures are prevalent.

## 2 Methods

### 2.1 Study design and participants

Data were collected in our neuroimaging laboratory in the Mirpur district of Dhaka, Bangladesh (Storrs, 2017). We recruited n = 210 infants aged 6-month (range 5.6–7.1 months) from the “Cryptosporidium Burden Study” (CRYPTO) and via door-to-door recruitment efforts in the Mirpur district of Dhaka. We also recruited n = 210 children aged 36-months (range 36.6–39.9 months) from the “Performance of Rotavirus and Oral Polio Vaccines in Developing Countries” (PROVIDE) study and door-to-door recruitment in Mirpur. Mothers of enrolled children completed a screening questionnaire which excluded children known to have been born preterm (<36 months gestation) or with perinatal complication. Children were also screened to ensure that no child had any known current neurological conditions, or physical or neurological disability. Finally, children were excluded if they were taking specific medications that may impact neural functioning and arousal. Mothers of the participating children provided informed, written consent for herself and the child to participate in the study. Ethical approval for the study was obtained from research review and ethics review committees at The International Centre for Diarrheal Disease Research, Bangladesh and Institutional Review Boards at Boston Children’s Hospital and were in accordance with local guidelines and regulations.

### 2.2 Baseline EEG data acquisition and processing

#### 2.2.1 Acquisition

Continuous EEG recordings were completed using a 128-Channel Hydrocel Sensor Net System (EGI, Inc., Eugene, OR, USA). Before application, the net was soaked in salt-water solution (1 L distilled water + 2 tsp potassium chloride (KCl) +5 mL Baby Shampoo) warmed to room temperature. The gains of the channels were checked during amplifier calibration before the EEG recording. Recordings were completed in dimly lit rooms with children sitting on the mother’s lap and facing a computer monitor positioned approximately 65 cm away. Trained staff were present during the recording and engaged minimally with the child during the recording, but would if necessary use a toy to re-direct children’s attention towards the screen. The EEG was recorded using a NetAmps 300 Amplifier with Cz being the online reference. Data were amplified and sampled at a frequency of 500 Hz while infants watched a video of an abstract screensaver accompanied by soothing sounds.

#### 2.2.2 Preprocessing

Raw EEG files were exported from NetStation 4.5.4 to MATLAB format (MathWorks, Inc). EEG recordings were offline filtered with an 8th order Butterworth band-pass (1–50 Hz) filter to remove the drift and line noise in the data. The filtered data were then segmented into 2 s epochs and inspected for artifacts using absolute algorithms (EEG > 100 μV or EEG < -100 μV), as well as independent component analysis for removing components related to eye movements, blinks, and focal activity (Xie et al., 2019b). Channel interpolation was conducted using a spherical spline interpolation with the EEGLAB function “eeg_interp” if there were fewer than 18 (15 %) electrodes that were missing or had bad data (Luyster et al., 2014; Xie et al., 2019c). After artifacts detection and rejection, EEG data were re-referenced to the average of all channels (i.e., common reference). To be comparable with prior studies on pediatric EEG power analysis, 20 standard 10–20 virtual electrodes were calculated with the five closest HGSN electrodes surrounding each 10–20 position (Fig. 2) (Pierce et al., 2019; Tomalski et al., 2013; Xie et al., 2018). Participants were excluded if they had less than 80 s clean data. A previous study using this dataset has shown that there was no effect of number of trials on EEG measures (Xie et al., 2019a). The final sample used for EEG analysis included 167 6-month-old infants and 187 36-month-old children.

#### 2.2.3 EEG power analysis

Power spectral densities (PSD) were calculated across all segments for each participant using Fast Fourier Transform (FFT) in Fieldtrip (Oostenveld et al., 2011). Absolute power was scaled in decibel (db)/Hz, i.e., 10*log10 (power) for four widely-studied frequency bands in the literature and age-appropriate boundaries were used to define them: theta (6 mos: 3–5 Hz; 36 mos; 3−6 Hz), alpha (6 mos: 6−9 Hz; 36 mos: 7 −10 Hz), beta (6 mos: 10–20 Hz; 36: 11−20 Hz), gamma (6 & 36 mos: 21–40 Hz). Regional EEG power was calculated for frontal, central, and parietal scalp regions. This was done by averaging across HGSN electrodes based on 10–20 positions, such that the electrodes surrounding F3, F4, and Fz were used to calculate the frontal EEG power, those surrounding the C3, C4, and Cz were used to calculate the central EEG power, and those surrounding P3, P4, and Pz were used to calculate the parietal EEG power. Relative power was also calculated for each region as the raw power in each frequency band divided by total power. To illustrate the PSD at each age, we created graphical plots that show the PSD over the full power spectrum.

### 2.3 Predictor variables

#### 2.3.1 Household wealth

Household wealth was assessed as a multi-dimensional construct following definitions of wealth in from the Demographic Health Surveys (DHS) and the “standards of living” component of the global multidimensional poverty indices defined by the Oxford Poverty and Human Development Initiative (Alkire et al., 2015). We used principal component analysis of data obtained from surveys, maternal interviews, and home observations. The principal components were created from 1) household income deciles created within this sample which spanned low and middle income families ; 2) a housing materials score that was based on a direct assessment conducted during home visits considering flooring materials, wall materials, roof materials, presence of cooking gas, toilet type, private versus shared toilet, open drain in from of house, municipality provided water supply, and crowding (>3 people per room) following DHS indicators ; 3) a count of household assets noted by an observer looking for presence of eight common assets: bed, table, chair, bench, almeria, phone, clock, ratio, television, bicycle, motor cycle, sewing machine, and a fan. See Table 1 for details. The unrotated PCA solution yielded three components of which only one had an eigenvalue higher than one (λ = 2.293). The proportion of variance explained by the first component was 77 %. All three indicators had loadings higher than 0.8 on the first component. Loadings for each indicator were 1 = 0.891; 2 = 0.890; 3 = 0.841. The score from this first component was used as the “household wealth” composite score in the analyses. Detailed output from the PCA is provided in the online supplement. There were no statistically significant differences between the household health scores in the 6 and 36 month-old samples [t(348) = 1.277, p = 0.202].

**Table 1:** Sample descriptive information.

	6-months old infants	36-months old children	Pearson’s chi square (count) or *t*-test comparison (continuous)
N	160	187	
Female, count (%)	86 (55.6 %)	77 (41.2 %)	Chi^2^ = 8.14, p < 0.017
Age in months, mean (SD)	6.60 (0.27)	37.27 (0.64)	Not applicable
Monthly income, taka (USD)	30111 ($355)	30089 ($355)	P > 0.001
Living below poverty line, count (%)*	89 (56 %)	111 (59 %)	P > 0.001
**Assets**, range 0−13, mean (SD)	6.9 (1.8)	6.7 (2.3)	P > 0.05
Have a phone, count (%)	97.5 %	89.3 %	
Have an almeria, count (%)	78.1 %	60.4 %	
Have a table, count (%)	46.2 %	52.4 %	
Have a chair, count (%)	60.6 %	61.0 %	
Have a bench, count (%)	10 %	8.6 %	
Have a clock, count (%)	80.6 %	77.5 %	
Have a bed, count (%)	97.5 %	98.4 %	
Have a radio, count (%)	0.6 %	3.7 %	
Have a television, count (%)	88.8 %	85.6 %	
Have a bicycle, count (%)	3.8 %	5.9 %	
Have a motor cycle, count (%)	5.0 %	8.0 %	
Have a sewing machine, count (%)	18.8 %	21.4 %	
Have a fan, count (%)	98.1 %	98.9 %	
**Housing risks,** range 0−9, mean (SD)	2.5 (1.7)	2.7 (2.0)	P > 0.05
Flooring: Earth (vs. cement, bamboo or wood)	1.2 %	5.3 %	
Walls: Tin, bamboo or straw (vs. cement or brick)	6.9 %	16.6 %	
Roof: Tin or straw (vs. finished roof)	45.6 %	55.6 %	
Has Cooking gas (vs. no gas)	23.8 %	21.4 %	
Toilet: Latrine or more primitive (vs. septic)	50.0 %	26.2 %	
Shared toilet (vs. private)	56.9 %	64.2 %	
Open drain in front of home (vs. none)	26.2 %	35.8 %	
No municipality supply	2.5 %	2.1 %	
Crowding (> 3 people pr. room)	40.0 %	40.6 %	
**Mother years of education**, range 0–10, mean (SD)	6.0 (3.5)	5.7 (3.9)	P > 0.05
**Maternal perceived stress**, range 0–40, mean (SD)	17.4 (7.4)	16.8 (7.1)	P > 0.05
Family Care Score**, range 0–14, mean (SD)	4.5 (1.8)	8.3 (2.5)	t(345)= −16.0, P < 0.001
Play activities with toys that make music	38.8 %	69.5 %	
Play activities involving drawing or writing	5.6 %	92.0 %	
Play activities pretending to be someone else	7.5 %	94.7 %	
Play activities encouraging movement	15.0 %	98.9 %	
Play activities teaching shapes and colors	16.2 %	23.0 %	
Reading activities	20.0 %	61 %	
Telling stories or nursery rhymes	65.6 %	73.8 %	
Singing	75.6 %	57.8 %	
Play activities with toys	85.0 %	70.1 %	
Counting or drawing	5.6 %	59.9 %	
Playing using fingers, arms and/or legs	65.6 %	55.6 %	
Chatting with child	48.8 %	78.1 %	
Child has access to books with pictures	11.9 %	74.9 %	
Child has access to magazines with pictures	25.0 %	39.6 %	

Binary variables reported as frequencies.

* Defined as of 2 dollars per day (161 taka) per person based on the household's monthly income and family size.

** Activities that a caregiver engaged in during the last 30 days.

#### 2.3.2 Maternal education

Maternal education was assessed based on maternal self-report using a sociodemographic questionnaire administered as an oral interview during the neurocognitive study visit. Maternal education was coded as completed years of education ranging from 0 to 10 years.

#### 2.3.3 Maternal perceived stress

Maternal perceived stress was assessed using the Perceived Stress Scale (Cohen et al., 1983), which was administered as an oral interview during the neurocognitive study visit. The Perceived Stress Scale includes 10 questions about stress and coping. Each item has likert-type response scale ranging from 0 =” never” to 4 = “very often”. All 10 items are summed to create a total score ranging from zero to 40. The Perceived Stress Scale had, to our knowledge, not previously been used in Bangladesh. We therefore went through a thorough process of translation, back-translation, and field testing before the start of data collection. The face validity of the Perceived Stress Scale was tested against a maternal depression score using Edinburgh Postnatal Depression Scale (Cox et al., 1987) and the Tension Scale, a scale developed specifically to assess mental health problems among South Asian women (Karasz et al., 2013). In the current sample, the perceived stress score was highly positively correlated with depression (r = 0.503) and tension (r = 0.546), and moderately negatively correlated with household wealth (r=-0.150). The Cronbach’s alpha was 0.642 in the 6 months old cohort and alpha = 0.604 in the 36 months old cohort indicating reasonable internal consistency.

#### 2.3.4 Family caregiving environment

The family caregiving environment was assessed using the play activity questions from the Family Care Indicators (Hamadani et al., 2010), which include items from UNICEF’s Multiple Indictor Cluster Survey (MISC Survey, 2021) to assess stimulating play activities that the mother, father, or another “caregiver” such as a grandparent engaged in with the child within the last 30 days. The scale assesses interactive activities include reading a book or looking at pictures in a book, telling stories to the child, singing a song or lullaby, taking the child outside the home, playing with the child, and naming, counting or drawing things as well as access to a book with pictures (yes/no) or magazine with pictures (yes/no). A full list of items included in the score is provided in Table 1. The Family Care Indicators have been widely used to assess the quality of children’s home environments in low and middle-income countries, including Bangladesh (Hamadani et al., 2010). We used a count of activities and materials the child was exposed to by the mother, father, or another caregiver. This score ranged from 0−15. As may be expected given the reliance of binary indicators Cronbach’s alpha was low in the younger cohort where activities were less commonly endorsed (alpha = 0.322) compared with the 36-month-old children were alpha = 0.641.

### 2.4 Statistical analyses

Statistical analyses were carried out using multivariate regression in StataSE version 15. Separate models were estimated for the 6 and 36-month-old infants and children, and for absolute and relative power. We first ran models with age, sex, household wealth, and maternal education as the independent variables. These models are referred to as Model 1 and results are provided in the online supplement. In a second step, we added the maternal stress and family care to the model to examine whether these social environmental factors independently accounted for variance in the EEG outcomes and/or changed the association of household wealth with EEG outcomes. These models are referred to as Model 2 and results are provided in Table 3, Table 4.

**Table 2:** Correlations among study variables.

		1	2	3	4	5	6	7	8	9	10	11	12	13	14	15	16	17
**6 months old infants**																		
1	Frontal Theta																	
2	Central Theta	0.851°																
3	Parietal Theta	0.812°	0.902°															
4	Frontal Alpha	0.583°	0.532°	0.422°														
5	Central Alpha	0.461°	0.598°	0.488°	0.866°													
6	Parietal Alpha	0.444°	0.532°	0.548°	0.793°	0.889°												
7	Frontal Beta	0.329°	0.306°	0.214**	0.611°	0.418°	0.331°											
8	Central Beta	0.424°	0.509°	0.383°	0.702°	0.697°	0.560°	0.751°										
9	Parietal Beta	0.399°	0.445°	0.495°	0.612°	0.609°	0.683°	0.590°	0.796°									
10	Frontal Gamma	0.155*	0.173*	0.089	0.460°	0.310°	0.235**	0.920°	0.637°	0.469°								
11	Central Gamma	0.235°	0.293**	0.204**	0.453**	0.423°	0.355°	0.669°	0.846°	0.655°	0.690°							
12	Parietal Gamma	0.204**	0.241**	0.268°	0.363°	0.327°	0.374°	0.561°	0.698°	0.811°	0.565°	0.851°						
13	Age	−0.003	−0.059	−0.082	0.084	0.083	0.076	−0.009	0.058	0.078	−0.042	0.046	0.048					
14	Sex	0.055	0.024	0.004	0.083	0.040	0.055	0.114	0.150	0.155*	0.154*	0.203*	0.164*	−0.009				
15	Wealth	−0.092	−0.051	−0.040	0.007	0.048	0.047	−0.001	0.060	0.118	−0.036	0.017	0.058	0.379°	−0.092			
16	Mat. education	−0.143	−0.088	−0.133	−0.036	−0.001	−0.036	−0.032	−0.025	−0.026	−0.055	−0.026	−0.042	0.176°	−0.119	0.543°		
17	Family Care	−0.011	0.007	0.011	0.017	0.044	0.040	−0.047	0.035	0.084	−0.072	0.057	0.084	0.155*	0.015	0.231**	0.209**	
18	Maternal Stress	−0.024	0.001	0.021	−0.037	−0.021	0.017	0.023	0.056	0.033	−0.002	0.034	0.040	−0.117	−0.040	−0.125	−0.018	−0.090

**36 months old children**																		
1	Frontal Theta																	
2	Central Theta	0.828°																
3	Parietal Theta	0.820°	0.924°															
4	Frontal Alpha	0.609°	0.652°	0.615°														
5	Central Alpha	0.510°	0.706°	0.640°	0.906°													
6	Parietal Alpha	0.444°	0.635°	0.648°	0.863°	0.939°												
7	Frontal Beta	0.506°	0.551°	0.459°	0.659°	0.589°	0.525°											
8	Central Beta	0.464°	0.672°	0.569°	0.761°	0.823°	0.756°	0.820°										
9	Parietal Beta	0.452°	0.645°	0.607°	0.734°	0.783°	0.771°	0.721°	0.928°									
10	Frontal Gamma	0.333°	0.354°	0.259°	0.369°	0.322°	0.279°	0.848°	0.549°	0.418°								
11	Central Gamma	0.334°	0.452°	0.337°	0.517°	0.521°	0.459°	0.756°	0.758°	0.625°	0.789°							
12	Parietal Gamma	0.388°	0.486°	0.410°	0.515°	0.515°	0.455°	0.646°	0.714°	0.755°	0.5856°	0.818°						
13	Age	0.112	0.129	0.134	0.174*	0.110	0.1131	0.174*	0.150*	0.168*	0.125	0.141	0.153*					
14	Sex	0.080	0.058	0.086	0.059	−0.031	−0.018	0.024	0.001	0.041	−0.040	−0.013	0.062	−0.002				
15	Wealth	−0.042	−0.076	−0.067	−0.058	−0.107	−0.098	−0.194**	−0.176*	−0.181*	−0.180*	−0.204**	−0.195**	0.258°	−0.017			
16	Mat. Edu.	−0.012	−0.015	0.016	−0.070	−0.066	−0.043	−0.130	−0.081	−0.065	−0.134	−0.098	−0.082	0.082	0.055	0.647°		
17	Family Care	−0.079	−0.021	−0.001	−0.020	−0.010	0.000	0.019	0.049	0.043	0.033	0.054	0.007	0.093	−0.025	0.201**	0.210**	
18	Maternal Stress	0.163*	0.148*	0.124	0.016	0.019	−0.012	0.096	0.032	0.006	0.114	0.044	0.080	−0.086	−0.034	−0.176*	−0.147*	−0.156*

* p < 0.05.

** p < 0.01.

° = < 0.001.

**Table 3:** Regression results from the 6 months old infants.

6 months absolute												
	Theta			Alpha			Beta			Gamma		
	Frontal	Central	Parietal	Frontal	Central	Parietal	Frontal	Central	Parietal	Frontal	Central	Parietal
**Child age**	0.010	−0.080	−0.109	0.074	0.055	0.045	−0.014	0.021	0.013	−0.039	0.023	0.003
	(0.572)	(0.550)	(0.593)	(0.565)	(0.686)	(0.636)	(0.678)	(0.486)	(0.486)	(0.771)	(0.534)	(0.528)
**Child sex**	0.042	0.019	−0.008	0.094	0.051	0.058	0.135	0.188*	0.168*	0.161*	0.224**	0.172*
	(0.288)	(0.277)	(0.299)	(0.285)	(0.345)	(0.320)	(0.341)	(0.245)	(0.245)	(0.388)	(0.269)	(0.266)
**Maternal education**	−0.130	−0.090	−0.168	−0.045	−0.034	−0.085	−0.034	−0.077	−0.121	−0.031	−0.039	−0.100
	(0.049)	(0.047)	(0.050)	(0.048)	(0.058)	(0.054)	(0.058)	(0.041)	(0.041)	(0.066)	(0.045)	(0.045)
**Wealth score**	−0.035	0.023	0.088	0.005	0.048	0.086	0.033	0.102	0.190	0.013	0.038	0.120
	(0.217)	(0.209)	(0.225)	(0.215)	(0.260)	(0.242)	(0.257)	(0.185)	(0.185)	(0.293)	(0.203)	(0.200)
**Family care**	0.024	0.045	0.061	0.008	0.034	0.040	−0.056	0.029	0.081	−0.073	0.059	0.089
	(0.081)	(0.078)	(0.084)	(0.080)	(0.097)	(0.090)	(0.096)	(0.069)	(0.069)	(0.109)	(0.075)	(0.075)
**Maternal stress**	−0.029	0.003	0.025	−0.017	0.010	0.054	0.013	0.069	0.070	−0.015	0.049	0.074
	(0.020)	(0.019)	(0.020)	(0.019)	(0.023)	(0.022)	(0.023)	(0.017)	(0.017)	(0.0264)	(0.018)	(0.018)
**N**	160	160	160	160	160	160	160	160	160	160	160	160
**R-sq**	0.025	0.014	0.032	0.017	0.011	0.017	0.023	0.048	0.066	0.035	0.058	0.053

6 months relative												
	Theta			Alpha			Beta			Gamma		
	Frontal	Central	Parietal	Frontal	Central	Parietal	Frontal	Central	Parietal	Frontal	Central	Parietal
**Child age**	−0.022	−0.162	−0.173*	0.107	0.118	0.128	−0.014	0.021	0.013	−0.046	0.049	0.029
	(0.662)	(0.530)	(0.552)	(0.207)	(0.340)	(0.320)	(0.678)	(0.486)	(0.486)	(0.060)	(0.028)	(0.028)
**Child sex**	−0.100	−0.094	−0.101	−0.036	−0.026	−0.001	0.135	0.188*	0.168*	0.123	0.199*	0.143
	(0.333)	(0.267)	(0.278)	(0.104)	(0.171)	(0.161)	(0.341)	(0.245)	(0.245)	(0.030)	(0.014)	(0.014)
**Maternal education**	−0.058	0.003	−0.019	0.034	0.017	0.043	−0.034	−0.077	−0.121	0.054	0.039	0.037
	(0.056)	(0.045)	(0.047)	(0.018)	(0.029)	(0.027)	(0.058)	(0.041)	(0.0413)	(0.005)	(0.002)	(0.002)
**Wealth score**	−0.044	−0.036	−0.014	0.028	0.024	−0.030	0.033	0.102	0.190	0.036	−0.009	0.046
	(0.251)	(0.201)	(0.210)	(0.078)	(0.129)	(0.122)	(0.257)	(0.185)	(0.185)	(0.023)	(0.011)	(0.011)
**Family care**	0.089	0.039	0.042	0.030	−0.053	−0.075	−0.056	0.029	0.081	−0.119	0.009	0.016
	(0.094)	(0.075)	(0.078)	(0.029)	(0.048)	(0.045)	(0.096)	(0.069)	(0.069)	(0.009)	(0.004)	(0.004)
**Maternal stress**	−0.009	−0.002	−0.032	0.017	−0.015	0.032	0.013	0.069	0.070	−0.015	0.023	0.022
	(0.023)	(0.018)	(0.019)	(0.007)	(0.012)	(0.011)	(0.023)	(0.017)	(0.017)	(0.002)	(0.001)	(0.001)
**N**	160	160	160	160	160	160	160	160	160	160	160	160
**R-sq**	0.021	0.038	0.041	0.022	0.020	0.020	0.023	0.048	0.066	0.030	0.042	0.027

Standardized beta coefficients; Standard errors in parentheses.

* p < 0.05.

** p < 0.01.

**Table 4:** Regression results from the 36-month-old children.

36 months absolute												
	Theta			Alpha			Beta			Gamma		
	Frontal	Central	Parietal	Frontal	Central	Parietal	Frontal	Central	Parietal	Frontal	Central	Parietal
**Child age**	0.146	0.173*	0.179*	0.201**	0.151	0.152*	0.243**	0.214**	0.237**	0.184*	0.212**	0.228**
	(0.196)	(0.213)	(0.225)	(0.236)	(0.302)	(0.284)	(0.200)	(0.204)	(0.191)	(0.273)	(0.187)	(0.170)
**Child sex**	0.080	0.056	0.082	0.060	−0.035	−0.025	0.023	−0.005	0.031	−0.038	−0.019	0.055
	(0.245)	(0.265)	(0.281)	(0.294)	(0.377)	(0.355)	(0.250)	(0.255)	(0.239)	(0.341)	(0.234)	(0.213)
**Maternal education**	0.056	0.086	0.128	−0.030	0.031	0.058	0.022	0.078	0.111	−0.004	0.081	0.101
	(0.042)	(0.045)	(0.0478)	(0.050)	(0.064)	(0.060)	(0.043)	(0.043)	(0.041)	(0.0579)	(0.040)	(0.036)
**Wealth score**	−0.074	−0.149	−0.177	−0.085	−0.166	−0.180	−0.267**	−0.292**	−0.326°	−0.223*	−0.323**	−0.314**
	(0.147)	(0.160)	(0.169)	(0.177)	(0.227)	(0.214)	(0.151)	(0.153)	(0.144)	(0.205)	(0.141)	(0.128)
**Family care**	−0.062	−0.000	0.014	−0.011	0.003	0.006	0.059	0.074	0.063	0.076	0.087	0.039
	(0.049)	(0.053)	(0.057)	(0.059)	(0.076)	(0.072)	(0.050)	(0.051)	(0.048)	(0.069)	(0.047)	(0.043)
**Maternal stress**	0.164*	0.151*	0.132	0.014	0.006	−0.022	0.082	0.022	−0.004	0.100	0.030	0.067
	(0.017)	(0.019)	(0.020)	(0.021)	(0.027)	(0.025)	(0.018)	(0.018)	(0.017)	(0.024)	(0.017)	(0.015)
**N**	187	187	187	187	187	187	187	187	187	187	187	187
**R-sq**	0.057	0.058	0.062	0.046	0.033	0.033	0.101	0.081	0.095	0.079	0.094	0.097

36 months relative												
	Theta			Alpha			Beta			Gamma		
	Frontal	Central	Parietal	Frontal	Central	Parietal	Frontal	Central	Parietal	Frontal	Central	Parietal
**Child age**	−0.124	−0.042	−0.021	0.067	0.018	−0.010	0.062	−0.002	−0.012	0.060	0.011	−0.023
	(0.147)	(0.147)	(0.150)	(0.103)	(0.152)	(0.142)	(0.017)	(0.015)	(0.0164)	(0.018)	(0.008)	(0.007)
**Child sex**	0.071	0.118	0.141	0.025	−0.090	−0.107	−0.014	0.008	0.012	−0.088	−0.036	0.015
	(0.184)	(0.183)	(0.187)	(0.129)	(0.189)	(0.177)	(0.021)	(0.019)	(0.0205)	(0.022)	(0.010)	(0.008)
**Maternal education**	0.074	0.020	0.028	−0.093	−0.023	−0.051	0.019	−0.008	−0.038	−0.039	−0.024	−0.067
	(0.031)	(0.031)	(0.032)	(0.022)	(0.032)	(0.030)	(0.004)	(0.003)	(0.003)	(0.004)	(0.002)	(0.001)
**Wealth score**	0.127	0.117	0.102	0.102	−0.042	−0.021	−0.195	−0.089	−0.078	−0.180	−0.123	−0.043
	(0.111)	(0.110)	(0.112)	(0.077)	(0.114)	(0.107)	(0.013)	(0.011)	(0.012)	(0.013)	(0.005)	(0.005)
**Family care**	−0.097	−0.015	−0.003	−0.003	−0.023	−0.026	0.110	0.075	0.057	0.092	0.077	0.004
	(0.037)	(0.037)	(0.038)	(0.026)	(0.038)	(0.036)	(0.004)	(0.002)	(0.004)	(0.004)	(0.002)	(0.002)
**Maternal stress**	0.086	0.142	0.128	−0.116	−0.088	−0.116	−0.061	−0.118	−0.133	0.009	−0.066	0.001
	(0.013)	(0.013)	(0.013)	(0.009)	(0.014)	(0.013)	(0.002)	(0.001)	(0.002)	(0.002)	(0.001)	(0.001)
**N**	187	187	187	187	187	187	187	187	187	187	187	187
**R-sq**	0.055	0.044	0.044	0.030	0.017	0.027	0.040	0.024	0.027	0.051	0.024	0.011

Standardized beta coefficients; Standard errors in parentheses.

* = p < 0.05.

** = p < 0.01.

° = p < 0.001.

Analyses included infants and children who had complete data (listwise deletion). We excluded two children (0.6 % of the total sample) who had missing information of child age and four children (1.1 % of the total sample) who had missing sociodemographic information. We also excluded one infant who was later found to be less than 5 months old at the time of data collection. This resulted in final sample sizes of 160 infants (aged 5.6–7.1 months) and 187 children (aged 36.6–39.9 months) with usable EEG data.

### 2.5 Additional datasets for contextualization of findings

To aid the interpretation of the findings, we examined cross-sectional developmental patterns in EEG power by plotting mean power across different age groups using data collected in 6, 24, 36, and 60 months old infants and children from Bangladesh (see below). All datasets were preprocessed and analyzed using the methods outlined above. The plots for the 6- and 36-month-old infants and children were made using data from the samples used in the current study. The 27-months plot was made using data collected during a follow-up assessment with a subset of the 6-month-old infants (n = 90) and the 60-months plot was made with data collected during a follow-up assessment with a subset of the 36-month-old children (n = 114).

## 3 Results

### 3.1 Descriptive sample information

Descriptive sample information and correlations among variables are provided in Table 1, Table 2, respectively. The mean monthly income across the sample was 355 USD and 56 % of the 6-month-old-infants compared with 59 % of the 36-month-old-infants lived below the World Bank poverty line defined as living on less than 2 USD per household member per day. With regard to maternal education, 85 % of the mothers of the 6-month-old infants, and 77 % of the mothers of the 36-month-old children had completed some level of schooling. The mean years of completed maternal education was 6.1 years in the 6-months old cohort and 5.7 years in the 36-months old cohort. There were no statistically significant differences between the two cohorts on any of the key variables expect for the family care indicators which were higher in the 36-month-old children as would be expected.

### 3.2 Association of EEG power with socioeconomic and psychosocial factors

The addition of maternal stress and the family care variables did not change the relationships of the other variables included in Model 1. We therefore provide the results from Model 2 in the tables. Results from Model 1 can be found in the supplemental material.

#### 3.2.1 The 6 month-old infants

We did not see any relationships between household wealth and either absolute or relative EEG power in the 6-month-old infants in Model 1. We also did not see any association of infant age or maternal education with absolute or relative EEG power. Child sex was associated with absolute EEG power such that female infants had higher absolute beta (central and parietal) and gamma power (frontal, central and parietal) compared with males. Child sex continued to be the only significant predictor of absolute EEG power after maternal stress and family care was added in Model 2. There were no associations of maternal stress or family care with EEG power.

#### 3.2.2 The 36-month-old children

In Model 1 we observed a negative association between household wealth and absolute EEG power in the beta and gamma bands across all three regions (frontal, central, parietal). We also observed a positive association between age and absolute EEG power in all four frequency bands and spanning all three regions, except for theta where the association only spanned frontal and central regions. We did not see any associations between absolute EEG power and child sex or maternal education. We also did not see any associations of the exposure variables with relative EEG power.

**Fig. 1:**
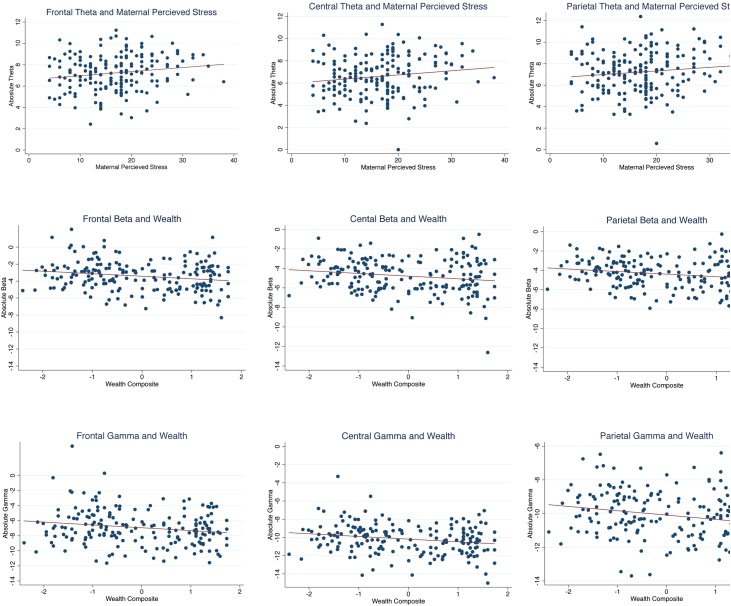
Scatter plots showing unadjusted bivariate correlations to illustrate significant relationships of regional EEG power with maternal stress and household wealth in the 36 month-old children.

Adding maternal stress and family care in Model 2 did not affect the relationship between household wealth and absolute power in the beta and gamma bands. Maternal stress was positively associated with absolute power in the theta band in the frontal and central regions. Family care did not show association with either absolute or relative EEG power. Fig. 1 illustrates bivariate correlation for significant associations.

### 3.3 Comparison of absolute and relative EEG power across 6 and 36-month-olds

**Fig. 2:**
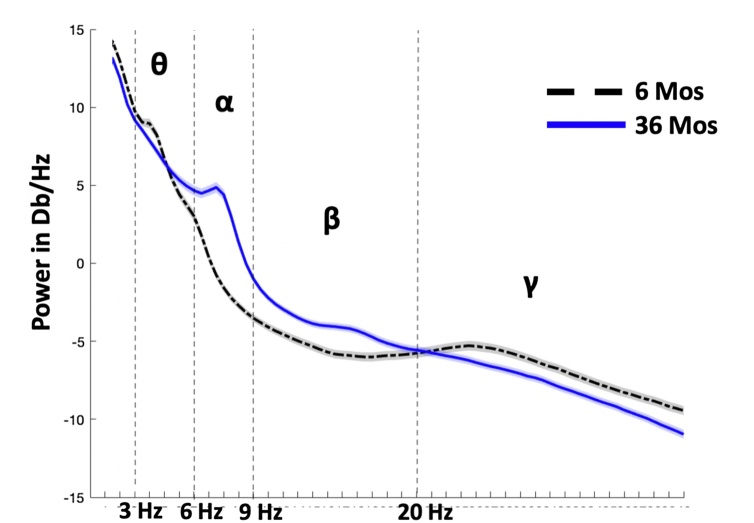
Power spectrum density (PSD) plots for the two cohorts. Illustration showing the global PSD plots for the two cohorts (6 and 36-month-olds). The shaded areas represent +/− 1 standard error. The plots show that 6-month-old infants (black, dotted) and 36-month-old children (blue, solid) exhibit different PSD patterns. The shade areas represent the 95 % confidence interval of the mean across the subjects, per group.

Fig. 2 shows PSD plots for absolute power for the 6-month-old infants (black, dotted) and 36-month-old children (blue, sold). The PSD plot for the 6-month-old infants shows a small peak in the theta band, whereas the PSD plot for the 36-month-old children shows a peak in the alpha band. These trends are in line with previously characterized developmental patterns of a high magnitude of low frequency power in infancy, which shifts towards higher frequencies (e.g. alpha) over development as described in the introduction discussed in the introduction. Of note, the observable decrease in gamma power from infancy to 36 months is likely due to a combination of functional changes in the brain and the increase of the skull thickness which reduces the detected signal.

**Fig. 3:**
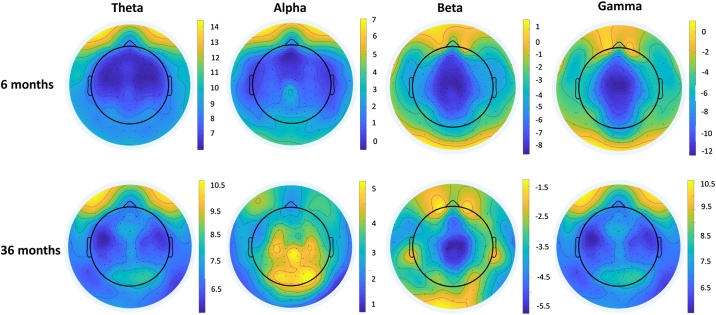
Topographical maps for EEG Power by Age and Frequency Band.

We also map differences in the scalp distribution of EEG power in the 6-month-old infants relative to the 36-month-old children. Fig. 3 shows topographical maps for the four frequency bands. The maps show that power in the theta, beta, and gamma bands have largely similar scalp distributions in the 6 and 36-month-olds. The scalp distribution of alpha power, however, appears to differ by age. In the 6-month-old infants, we see that alpha activity is most prominent in the frontal area, whereas for the 36-month-old children we find that alpha activity is most prominent in the central and parietal regions, a pattern that resembles that observed in adults.

### 3.4 Cross-sectional illustration of EEG power over development using multi-cohort data

**Fig. 4:**
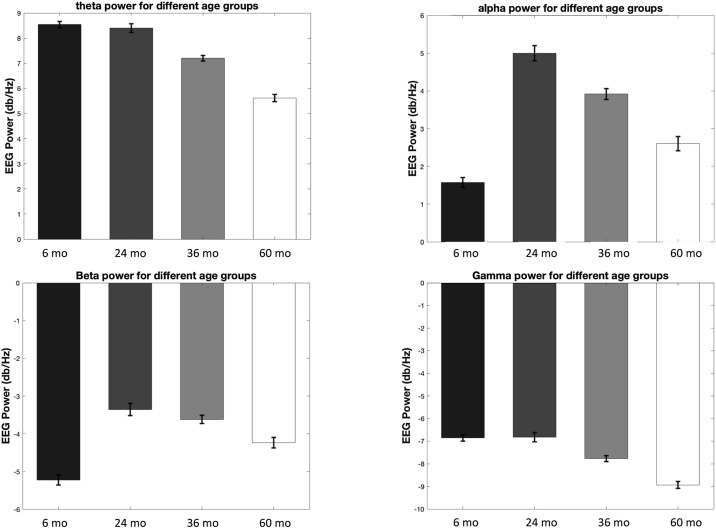
Mean log10 transformed absolute power in different frequencies bands across different age groups of children recruited from studies within the Nelson Laboratory in Bangladesh at ages 6, 24, 36 and 60 months [m]. The 24- (N = 90) and 60-month-old (N = 114) cohorts were longitudinal samples for the 6- and 36-month-old cohorts respectively. Data collection and processing is still ongoing for these two longitudinal age groups (i.e., 24 and 60 months), and their EEG data were only included in this figure to show developmental changes of EEG power but not included in the main analyses of the study.

Multi-timepoint data was plotted to illustrate developmental patterns of spectral EEG power across a broader age range from 6 to 60 months in an effort to contextualize findings from the current study and aid interpretation of results. These PSD plots are illustrated in Fig. 4. Cohort information is included in the legend. A number of patterns emerged. For the theta band, the PSD was found to be similar in between 6- and 24 and 36-month-olds, but lower in 36 and 60-month-olds. More variation with age was observed in the alpha band where the alpha peak frequency was foundto be gradually shifted towards higher frequencies with age. The 6-month-old infants, on average, show lower levels of alpha power compared with older children. The developmental pattern observed in the alpha band are consistent with what has previously shown among children from the US (Marshall et al., 2002). Beta power is lowest in the 6-month-olds, intermediate in the 60-month-olds, and highest in the 24- and 36-month-month-old children. Such pattern seems to suggest a complex, possibly inverted U-shaped trajectory of change in beta power throughout early childhood, as opposed to a linear shift. For gamma power, we found that the PSD was roughly similar between 6 and 24 months of age, but was progressively lower in 36- and 60-month-old children, suggesting a negative linear trend.

## 4 Discussion

In this study we tested two central hypotheses: 1) that household wealth is positively associated with absolute high-frequency EEG power in 6-month-old infants and 36-month-old children, but possibly more strongly so in 36-month-old children given prolonged exposure to environmental factors; 2) and that the association between household wealth and spectral EEG power is partially accounted for by psychosocial factors related to maternal stress and caregiving activities. Our results did not show any associations of household wealth, maternal stress, or family care with EEG power in the 6-month-old infants. The finding of no association of EEG power with socioeconomic or psychosocial factors in the 6-month-old infants is similar to the absence of association observed in neonates in the United States (Britto et al., 2016). It deviates, however, from findings of association in 2–9 months old infants from the US and UK. For example, Pierce et al. (2019) reported a negative association of maternal perceived stress with EEG power in the beta and gamma bands, and a positive relationship between maternal education and absolute gamma power in 2-month-old infants from the United States (Pierce et al., 2019). Similarly, Troller-Renfree et al. (2020) found that maternal physiological stress (hair cortisol) was positively associated with relative theta power and negatively associated with relative beta power in 6 to 12-month-old infants. Finally, Tomalski et al. (2013) have found that household income was positively associated with absolute gamma power in 6–9-month-old infants in the United Kingdom.

The absence of association in the 6-month-old infants enrolled in this study is largely in line with behavioral findings from the Crypto cohort who constitute more than half of the sample of the 6-month-olds included in this study. More specifically, we have previously shown that household wealth did not predict developmental milestones assessed on Mullen Scales of Early Learning in 6-month-olds, although we did see a selective association of family care with receptive language at 6 months (Jensen et al., 2019c).

A possible reason for the absence of association of wealth, maternal stress, and family care with EEG power in the 6 months old infants in the current study could be insufficient variation across predictor variables to detect subtle neurofunctional effects. Indeed, a prior study of these children similarly failed to find an association between EEG functional connectivity and environmental factors including household wealth and family caregiving in the 6 months old infants either (Xie et al., 2019a). The degree of risk exposure and amount of variance captures across the predictors is, however, largely similar in the 6 and 36-month-old cohorts so methodological effects do not seem to explain this apparent difference in trends across the two age groups. While the questionnaires used to assess household wealth and family care draw on items from international surveys commonly used in low and middle income countries, we note that the perceived stress scale was developed in a western context and although the concurrent validity indicated association of maternal perceived stress with related measures such as depression cultural factors may affect our ability to capture maternal perceived stress. The mean levels of maternal stress were moderate (17.4 in mothers of the 6 month-old infants and 16.8 in mothers of the 36 month-old children) and comparable with means of 16.8 among Chinese mothers of 6–8 week old infants (Gao et al., 2009) and 22.4–23.4 among Ugandan mothers aged 14–46 (Goldstein et al., 2018). Interestingly, although the mean levels of perceived stress in the current study were lower than the mean levels of 20.5 reported in American mothers of six to 12 month-old infants in a study that did not find any association between maternal perceived stress and infant EEG (Troller-Renfree et al., 2020), perceived stress rates were markedly higher than the mean score of 10.2 and 12.9 observed among American mothers of 2 month-old infants in a study that did find association of maternal stress and infant EEG power (Pierce et al., 2019).

We did not observe any neural correlates of the exposure variables in the 6-month-olds but we did find association of both household wealth and maternal perceived stress in the 36-month-old children. More specifically, we observed a negative association between household wealth with absolute beta and gamma power in the 36-month-old children. This association was sustained when maternal stress and family care were added to the model suggesting that the association of wealth on neural functioning is independent of psychosocial factors. In the 36-month-old children we also found that higher levels of maternal stress were associated with more absolute power in the theta band. We did not find any associations of family care with EEG power at in either the 6 or 36 month-olds. The finding of a relationship between household wealth and EEG power in the 36-month-old children is in line with our hypothesis of association, yet the direction of the association was in the opposite direction to what we hypothesized. That is to say that we expected household wealth to be positively correlated with absolute high frequency power (i.e., what would normally be considered a “mature” EEG power profile), yet we found a negative association between household wealth and absolute power across the high frequency bands (beta and gamma). Our hypothesis was based on studies from the United Kingdom and the United States (Pierce et al., 2019; Tomalski et al., 2013; Brito et al., 2016; Troller-Renfree et al., 2020) and Mexico (Otero, 1994, 1997), which generally have reported negative associations between children’s socioeconomic risks (low wealth and low maternal education) and high frequency EEG power. Such a pattern has previously been taken to support a “developmental delay hypothesis” whereby low levels of high frequency power reflects an “immature” EEG power profile that may be caused by a delay in the expected developmental shift from predominance of low frequency power towards more high frequency power during development (Saby and Marshall, 2012). To aid the interpretation of current findings within the context of neurodevelopment change, we used data from four different age groups of children in Bangladesh to do a cross-sectional examination of developmental changes in spectral EEG power. The patterns that emerged suggest that beta power does not increase linearly over development, but follows a more complex, possibly inverted U-shaped trajectory of change from infancy through early childhood. Moreover, we observed a negative developmental trend for gamma power starting around 24 months of age. If high frequency beta and gamma power starts to decrease around the age of 24–36 months, then the negative association of household wealth with beta and gamma power may indicate that more wealth is associated with a more “mature” EEG power profile.

Several factors may explain discrepancies between current and previous findings. First, as evident from our review of the literature, most previous studies of socioeconomic and psychosocial influences on EEG power have been limited to infants ranging in age from neonates to 12-month-olds. Developmental patterns and the possible susceptibility of neural development to environmental insults is therefore not well-characterized in older children. While we based our hypothesis of a positive association between household wealth and high frequency EEG power on an assumption of a positive linear relationship between wealth and high frequency EEG power across development, current findings indicate that the idea of a mature EEG profile as characterized by more high frequency relative to low frequency power may be too simplistic. Large-scale longitudinal studies of normative developmental changes in EEG are therefore warranted. Future research should also examine how socio-economic and psychosocial factors affect developmental change in EEG power to supplement cross-sectional patterns shown here. A second possible explanation for the different direction of the association of wealth with high frequency power is that the circumstances in which children grow up in a poor neighborhood in a low-income country are very different from those of a poor neighborhood in a high income country. For example, extreme poverty is often characterized by more severe compound exposures due to extreme crowding and poor sanitation (See Table 1 for prevalence rates of different risks in the current sample). These complex environmental risk exposures may influence neural development differently from observed associations of poverty with neurodevelopmental outcomes in high income countries. For example, some families enrolled in this study lived in extreme poverty (yearly income of $210) still, the median yearly income was $1488 in the 6-month-olds and $1134 in the 36-month-olds which is close to the average per capita income in Bangladesh which was $1,564 in 2017. around the time when the data for the current study was collected (World Bank Data) reflecting the fact that the current sample spans poor and middle class households in Dhaka. The per capita income in Bangladesh and in this sample is, however, much lower than the average global per capita of $10,828 in 2017 and the per capita income of $60,062 in the United States where most comparison studies took place. The median income in Bangladesh and this sample is, however, considerably higher than many other low income countries that have a per capita income less than $800. Histograms showing the monthly income distribution for the 6- and 36 month old households are provided in the supplemental material.

As noted above, the families enrolled in the current study live in urban and extremely densely crowded neighborhoods: the population density in Dhaka is estimated at 23,234 people per square kilometer compared with 5,344 people per square kilometer in Boston (World Population Review, 2016). Moreover, the homes of enrolled families typically consist of one single room with a shared bathroom and shared kitchen containing a simple gas stove and a sink. All homes, however, had electricity, the far majority (85–88 %) of the homes had a television and, among the 36 month-olds, 75 % of the children had access to books with pictures. The high rates of playful interactions captured by the family care indicators, and the finding that more than >70 % of parents report that a caregiver has played with the infant/ child with toys also suggests that the children enrolled in the current study live in overall stimulating home environments. Notably, the prevalence of caregiver engagement in play with toys is much higher than the 37 % of caregivers who reported playing with children with toys in a study of 18 month-old children in rural Bangladesh (Hamadani et al., 2010) but similar to findings from UNICEF’s Multiple Indicator Survey from Bangladesh from 2019 which found that 86 % of children under 5 years from urban areas had manufactured toys. Clearly, poverty looks different in different settings, highlighting the importance of culturally sensitive and holistic approaches to child development research.

From a neurodevelopmental perspective we note that there may be instances where high levels of spectral power in certain frequency bands may interfere with optimal neural functioning. We offer two neurodevelopmental explanations for processes that may explain why EEG power may be negatively associated with wealth and positively associated with socioeconomic and psychosocial risk exposures. These involve “increased myelination stemming from living in an *overstimulating* environment” and “delayed or attenuated inhibitory regulation of neural responses”. The first explanation builds on the hypothesis that poverty and psychosocial stress may result in high levels of EEG power through the influence of stress on the myelination of neurons. Myelination occurs rapidly over the first few years of life and serves to increase conduction velocity and synaptic efficiency (Paus, 2010). Although poverty is often considered a proxy for resource deprivation (e.g., inadequate supply of nutrition, health care, child care resources, etc.) poverty may, in certain situations, manifest as an “overstimulating” environment characterized by crowding, loud noises, etc. Such conditions (widely observed in Dhaka) may lead to accelerated myelination which, in turn, may lead to increased spectral EEG power. The second explanation builds on the fact that the net sum of neuronal activity is determined by the balance of concurrent exhibitory and inhibitory neural processes. Different neuropathological conditions including Autism Spectrum Disorders (Nelson and Valakh, 2015), schizophrenia (Gao and Penzes, 2015), and Rett Syndrome (Dani et al., 2005) have been proposed to involve an imbalance of excitatory and inhibitory cerebral signaling, that are believed to contribute to neurofunctional disturbances. Although the actual neurological mechanism is not fully understood, it likely involves a combination of genetic and environmental factors that affect GABAergic control of neurons (Gao and Penzes, 2015) and could result in excessive absolute power.

Key strengths of this paper are the large sample size and the uniqueness of the population that is highly underrepresented in neurodevelopmental research. Key limitations are the use of cross-sectional as opposed to longitudinal data and inability to account for exposure to malnutrition and infectious disease, both of which are common risks among children growing up in global poverty. While the cited comparison studies examining associations between wealth-related risks and maternal stress with infant EEG similarly did not account for malnutrition these studies typically exclude children with major medical problems which in most instances would include malnutrition. The rate of medical risks is therefore considerably higher in this study given high rates of malnutrition and high frequency of infectious disease such as diarrhea. We did, exclude children who exhibited acute symptoms of illness, still infants and children enrolled in this study are likely to have had greater *life-time* exposure to medical problems than infant and children included in previous EEG studies of similar nature.

Future studies should examine association of infant health including long-term and acute malnutrition with EEG power since both are known to influence neural growth (Prado and Dewey, 2014). Future studies should also examine predictive associations of EEG power with behavioral outcomes to understand the possible functional implications of observed neurological effects. For example, while Brito et al. (2016) did not find any association of socioeconomic risks with EEG power in neonates, they did find that EEG power at birth predicted later visual memory and language scores. Moreover, Cantiani et al. (2019) found that gamma power at 6 months mediated the effect of socioeconomic status on language scores at 24 months.

## 5 Conclusion

This study found evidence for association of household wealth with spectral EEG power in 36-month-old children living in an urban low to middle income neighborhood in Dhaka, Bangladesh. More specifically, we found that household wealth was negatively associated with absolute EEG power in two high frequency bands, namely beta, and gamma. We also found that maternal stress was positively associated with absolute theta power in 36-month-old children. We did not see any associations of either household wealth or maternal stress with spectral EEG power in the 6-month-old infants. We also did not see any association of family caregiving activities with EEG power in either the 6 or 36-month-olds. Taken together the current findings seem to suggest that environmental factor related to socioeconomic status and maternal stress influence neural signaling, but that such effects emerge sometime between 6 and 36 months of age.

In line with previous research the current findings support the idea that EEG may serve as a method for detection of neural correlates of early life risk exposures. As such, EEG may help to identify children who stand to benefit from early intervention to alleviate risks (for example, by stratifying the sample by risk status inferred from EEG). Still, more research is needed to characterize expected developmental patterns of change in spectral EEG to support the interpretation results. Existing knowledge of developmental trajectories in resting state EEG comes from predominantly small cross-sectional studies. Future longitudinal studies are needed to characterize developmental changes in resting state spectral power across different environments, including populations from low and middle income countries where little is known about typical and atypical neurodevelopmental processes.

## Data available

Data will be available upon request

## Author contributions

**Sarah K. G. Jensen:** Conceptualization of project, Methodology, Formal analysis, Writing - Original Draft, Visualization; **Wanze Xie:** Methodology, Visualization, Writing – review & editing; Swapna Kumar: Project administration, Data Curation; **Rashidul Haque:** Project administration; **William A. Petri Jr.:** Methodology, Funding acquisition; **Charles A. Nelson III:** Conceptualization of project; Methodology, Funding acquisition, Supervision, Resources, Writing- Reviewing and Editing.

## Funding source

Funding for the study was provided by research grants from the Bill & Melinda Gates Foundation to CAN [OPP1111625] and WAP [OPP1017093], and research grants to WAP from the Henske Family and the 10.13039/501100012264NIH (R01 AI043596). The funders had no role in study design, data analysis or writing of the manuscript.

## Declaration of Competing Interest

The authors declare that they have no known competing financial interests or personal relationships that could have appeared to influence the work reported in this paper.
