# Supplementary material for: Associations of socioeconomic and other environmental factors with early brain development in Bangladeshi infants and children
Source: Dev Cogn Neurosci. 2021 Jun 18;50:100981. doi: 10.1016/j.dcn.2021.100981 (PMC8254021; doi:10.1016/j.dcn.2021.100981)
Supplement: Supplementary file 1 [file mmc1.docx]

SUPPLEMENTAL MATERIAL FOR:

Associations of socioeconomic and other environmental factors with early brain development in Bangladeshi infants and children

Sarah K. G. Jensen^1,2,3^, Wanze Xie^1,2^, Swapna Kumar^1^, Rashidul Haque^4^, William A. Petri^5^, Charles A. Nelson III^1,2,6^

^1^ Department of Pediatrics, Boston Children’s Hospital, Boston, Massachusetts, USA

^2^ Department of Pediatrics, Harvard Medical School, Boston, Massachusetts, USA

^3^ Boston College School of Social Work, Boston College, Massachusetts, USA

^4^ Icddr,b, Dhaka, Bangladesh

^5^ University of Virginia, Infectious Diseases & International Health, Charlottesville, Virginia, USA

^6^ Harvard Graduate School of Education, Cambridge, Massachusetts, USA

**Supplemental text**

Factor analysis/correlation Number of obs = 350

Method: principal-component factors Retained factors = 1

Rotation: (unrotated) Number of params = 3

--------------------------------------------------------------------------

Factor | Eigenvalue Difference Proportion Cumulative

-------------+------------------------------------------------------------

Factor1 | 2.29340 1.87010 0.7645 0.7645

Factor2 | 0.42330 0.13999 0.1411 0.9056

Factor3 | 0.28330 . 0.0944 1.0000

--------------------------------------------------------------------------

LR test: independent vs. saturated: chi2(3) = 449.44 Prob>chi2 = 0.0000

Factor loadings (pattern matrix) and unique variances

---------------------------------------

Variable | Factor1 | Uniqueness

-------------+----------+--------------

income_dec | 0.8907 | 0.2067

housing_re~e | 0.8904 | 0.2072

Assets_pos~r | 0.8410 | 0.2928

---------------------------------------

(blanks represent abs(loading)<.3)

Scoring coefficients (method = regression)

------------------------

Variable | Factor1

-------------+----------

income_dec | 0.38837

housing_re~e | 0.38825

Assets_pos~r | 0.36669

------------------------

**Output from the PCA analysis constructing the household wealth**

**Supplemental Figures**

**Supplemental Table S1:** **Regression results from the 6 months old infants**

| **6 months absolute** | | | | | | | | | | | | |
| --- | --- | --- | --- | --- | --- | --- | --- | --- | --- | --- | --- | --- |
|  | **Theta** | | | **Alpha** | | | **Beta** | | | **Gamma** | | |
|  | **Frontal** | **Central** | **Parietal** | **Frontal** | **Central** | **Parietal** | **Frontal** | **Central** | **Parietal** | **Frontal** | **Central** | **Parietal** |
| **Child age** | 0.015 | -0.076 | -0.105 | 0.076 | 0.057 | 0.045 | -0.020 | 0.019 | 0.016 | -0.044 | 0.025 | 0.006 |
|  | (0.565) | (0.544) | (0.587) | (0.559) | (0.678) | (0.630) | (0.671) | (0.482) | (0.483) | (0.763) | (0.529) | (0.524) |
| **Child sex** | 0.044 | 0.021 | -0.006 | 0.095 | 0.052 | 0.058 | 0.131 | 0.187* | 0.170* | 0.158* | 0.225** | 0.174* |
|  | (0.285) | (0.275) | (0.297) | (0.282) | (0.342) | (0.318) | (0.339) | (0.243) | (0.244) | (0.386) | (0.267) | (0.265) |
| **Maternal education** | -0.028 | 0.028 | 0.092 | 0.008 | 0.051 | 0.083 | 0.024 | 0.095 | 0.189 | 0.006 | 0.037 | 0.120 |
|  | (0.213) | (0.205) | (0.222) | (0.211) | (0.256) | (0.238) | (0.253) | (0.182) | (0.182) | (0.288) | (0.200) | (0.198) |
| **Wealth score** | -0.128 | -0.084 | -0.159 | -0.045 | -0.029 | -0.077 | -0.040 | -0.069 | -0.107 | -0.041 | -0.029 | -0.084 |
|  | (0.0480) | (0.0461) | (0.0498) | (0.0474) | (0.0575) | (0.0534) | (0.0569) | (0.0409) | (0.0410) | (0.0648) | (0.0449) | (0.0445) |
| **N** | 160 | 160 | 160 | 160 | 160 | 160 | 160 | 160 | 160 | 160 | 160 | 160 |
| **R-sq** | 0.024 | 0.013 | 0.028 | 0.017 | 0.009 | 0.013 | 0.019 | 0.043 | 0.056 | 0.030 | 0.053 | 0.041 |
| **6 months relative** | | | | | | | | | | | | |
|  | **Theta** | | | **Alpha** | | | **Beta** | | | **Gamma** | | |
|  | **Frontal** | **Central** | **Parietal** | **Frontal** | **Central** | **Parietal** | **Frontal** | **Central** | **Parietal** | **Frontal** | **Central** | **Parietal** |
| **Child age** | -0.013 | -0.158 | -0.167 | 0.109 | 0.114 | 0.119 | -0.046 | 0.066 | 0.100 | -0.056 | 0.048 | 0.029 |
|  | (0.657) | (0.524) | (0.546) | (0.204) | (0.337) | (0.318) | (0.0505) | (0.0331) | (0.0341) | (0.0606) | (0.0280) | (0.0280) |
| **Child sex** | -0.095 | -0.091 | -0.097 | -0.035 | -0.028 | -0.006 | 0.085 | 0.159* | 0.171* | 0.118 | 0.198* | 0.143 |
|  | (0.332) | (0.265) | (0.276) | (0.103) | (0.170) | (0.160) | (0.0255) | (0.0167) | (0.0172) | (0.0306) | (0.0141) | (0.0141) |
| **Maternal education** | -0.031 | -0.031 | -0.003 | 0.029 | 0.020 | -0.044 | 0.037 | 0.080 | 0.120 | 0.024 | -0.011 | 0.045 |
|  | (0.248) | (0.198) | (0.206) | (0.0770) | (0.127) | (0.120) | (0.0191) | (0.0125) | (0.0129) | (0.0229) | (0.0106) | (0.0106) |
| **Wealth score** | -0.048 | 0.008 | -0.015 | 0.038 | 0.010 | 0.036 | 0.051 | -0.004 | 0.049 | 0.039 | 0.042 | 0.041 |
|  | (0.0557) | (0.0445) | (0.0464) | (0.0173) | (0.0286) | (0.0269) | (0.00429) | (0.00281) | (0.00289) | (0.00514) | (0.00238) | (0.00238) |
| **N** | 160 | 160 | 160 | 160 | 160 | 160 | 160 | 160 | 160 | 160 | 160 | 160 |
| **R-sq** | 0.013 | 0.037 | 0.038 | 0.021 | 0.017 | 0.013 | 0.012 | 0.038 | 0.068 | 0.017 | 0.042 | 0.026 |

Supplemental Table S1: **Regression results from the 6 months old infants**

| **36 months absolute** | | | | | | | | | | | | |
| --- | --- | --- | --- | --- | --- | --- | --- | --- | --- | --- | --- | --- |
|  | **Theta** | | | **Alpha** | | | **Beta** | | | **Gamma** | | |
|  | **Frontal** | **Central** | **Parietal** | **Frontal** | **Central** | **Parietal** | **Frontal** | **Central** | **Parietal** | **Frontal** | **Central** | **Parietal** |
| **Child age** | 0.134 | 0.166* | 0.173* | 0.199** | 0.151* | 0.154* | 0.242** | 0.217** | 0.240** | 0.184* | 0.216** | 0.227** |
|  | (0.198) | (0.213) | (0.226) | (0.234) | (0.300) | (0.282) | (0.200) | (0.203) | (0.190) | (0.273) | (0.187) | (0.170) |
| **Child sex** | 0.076 | 0.051 | 0.077 | 0.060 | -0.035 | -0.024 | 0.018 | -0.008 | 0.029 | -0.044 | -0.023 | 0.051 |
|  | (0.247) | (0.267) | (0.282) | (0.293) | (0.374) | (0.353) | (0.250) | (0.254) | (0.238) | (0.341) | (0.233) | (0.212) |
| **Maternal education** | 0.037 | 0.077 | 0.122 | -0.033 | 0.031 | 0.060 | 0.026 | 0.088 | 0.121 | 0.002 | 0.092 | 0.102 |
|  | (0.0418) | (0.0450) | (0.0476) | (0.0494) | (0.0632) | (0.0596) | (0.0421) | (0.0428) | (0.0401) | (0.0576) | (0.0394) | (0.0358) |
| **Wealth score** | -0.100 | -0.168 | -0.192 | -0.087 | -0.166 | -0.177 | -0.272** | -0.288** | -0.320** | -0.229* | -0.319** | -0.319** |
|  | (0.148) | (0.160) | (0.169) | (0.175) | (0.224) | (0.211) | (0.150) | (0.152) | (0.142) | (0.204) | (0.140) | (0.127) |
| **N** | 187 | 187 | 187 | 187 | 187 | 187 | 187 | 187 | 187 | 187 | 187 | 187 |
| **R-sq** | 0.025 | 0.036 | 0.045 | 0.046 | 0.033 | 0.033 | 0.092 | 0.076 | 0.091 | 0.066 | 0.087 | 0.092 |
| **36 months relative** | | | | | | | | | | | | |
|  | **Theta** | | | **Alpha** | | | **Beta** | | | **Gamma** | | |
|  | **Frontal** | **Central** | **Parietal** | **Frontal** | **Central** | **Parietal** | **Frontal** | **Central** | **Parietal** | **Frontal** | **Central** | **Parietal** |
| **Child age** | -0.133 | -0.050 | -0.027 | 0.072 | 0.021 | -0.006 | 0.072 | 0.008 | -0.003 | 0.065 | 0.019 | -0.023 |
|  | (0.147) | (0.147) | (0.150) | (0.103) | (0.151) | (0.142) | (0.0170) | (0.0149) | (0.0165) | (0.0178) | (0.00789) | (0.00672) |
| **Child sex** | 0.071 | 0.114 | 0.137 | 0.029 | -0.086 | -0.102 | -0.015 | 0.009 | 0.014 | -0.091 | -0.036 | 0.014 |
|  | (0.184) | (0.184) | (0.187) | (0.129) | (0.189) | (0.177) | (0.0213) | (0.0187) | (0.0206) | (0.0222) | (0.00986) | (0.00840) |
| **Maternal education** | 0.054 | 0.009 | 0.020 | -0.087 | -0.021 | -0.048 | 0.039 | 0.010 | -0.021 | -0.026 | -0.008 | -0.066 |
|  | (0.0311) | (0.0310) | (0.0316) | (0.0217) | (0.0319) | (0.0299) | (0.00359) | (0.00315) | (0.00347) | (0.00375) | (0.00166) | (0.00142) |
| **Wealth score** | 0.108 | 0.098 | 0.086 | 0.116 | -0.034 | -0.009 | -0.177 | -0.067 | -0.057 | -0.173 | -0.107 | -0.043 |
|  | (0.110) | (0.110) | (0.112) | (0.0770) | (0.113) | (0.106) | (0.0127) | (0.0112) | (0.0123) | (0.0133) | (0.00591) | (0.00503) |
| **N** | 187 | 187 | 187 | 187 | 187 | 187 | 187 | 187 | 187 | 187 | 187 | 187 |
| **R-sq** | 0.037 | 0.023 | 0.028 | 0.017 | 0.010 | 0.014 | 0.023 | 0.004 | 0.005 | 0.043 | 0.013 | 0.011 |
